# Effective synthesis of circRNA via a thermostable T7 RNA polymerase variant as the catalyst

**DOI:** 10.3389/fbioe.2024.1356354

**Published:** 2024-04-09

**Authors:** Wei He, Xinya Zhang, Yangxiaoyu Zou, Ji Li, Le Chang, Yu-Cai He, Qiuheng Jin, Jianren Ye

**Affiliations:** ^1^ College of Biology and the Environment, Nanjing Forestry University, Nanjing, China; ^2^ Vazyme Biotech Co., Ltd, Nanjing, China; ^3^ School of Pharmacy, Changzhou University, Changzhou, China

**Keywords:** circRNA, transcription, thermostability, mutations, cyclization, synthesis

## Abstract

**Introduction:**

Circular RNAs (circRNAs) are endogenous noncoding RNAs (ncRNAs) with transcriptional lengths ranging from hundreds to thousands. circRNAs have attracted attention owing to their stable structure and ability to treat complicated diseases. Our objective was to create a one-step reaction for circRNA synthesis using wild-type T7 RNA polymerase as the catalyst. However, T7 RNA polymerase is thermally unstable, and we streamlined circRNA synthesis via consensus and folding free energy calculations for hotspot selection. Because of the thermal instability, the permuted intron and exon (PIE) method for circRNA synthesis is conducted via tandem catalysis with a transcription reaction at a low temperature and linear RNA precursor cyclization at a high temperature.

**Methods:**

To streamline the process, a multisite mutant T7 RNA polymerase (S430P, N433T, S633P, F849I, F880Y, and G788A) with significantly improved thermostability was constructed, and G788A was used.

**Results:**

The resulting mutant exhibited stable activity at 45°C for over an hour, enabling the implementation of a one-pot transcription and cyclization reaction. The simplified circRNA production process demonstrated an efficiency comparable to that of the conventional two-step reaction, with a cyclization rate exceeding 95% and reduced production of immunostimulatory dsRNA byproducts.

## 1 Introduction

Circular RNAs (circRNAs) are naturally occurring endogenous noncoding RNAs (ncRNAs) with transcriptional lengths ranging from hundreds to thousands of ribonucleotides. The group was first discovered in 1976 while studying plant viroids (Sanger et al., 1976). circRNAs indirectly regulate and participate in the expression of miRNA targets in various physiological and pathological pathways (Zhang et al., 2013; Li et al., 2015). In recent years, circRNAs have been implicated in various diseases, including cancer (Wesselhoeft et al., 2018; Li J. et al., 2020; Li R. et al., 2020; Chen and Shan, 2021; Zhu et al., 2021; Liu et al., 2022), cardiovascular diseases (Altesha et al., 2019; Su and Lv, 2020; Tang et al., 2021; Ju et al., 2022), and neurodegenerative disorders (Jiao et al., 2023). The application of circRNAs has attracted widespread attention among drug researchers, owing to their stable structure and ability to cure complicated diseases (Zhu et al., 2021; Lee et al., 2022; Zhao et al., 2022). However, circRNA research is still in its early stages, and the synthesis of circRNA as a drug has become a research hotspot.

Typically, two-step linear RNA precursor transcription and cyclization are involved in the synthesis of circRNAs. Like mRNA synthesis, linear RNA precursors are transcribed by RNA polymerases (RNAP) *in vitro* (Pardi et al., 2018; Deng et al., 2022; Rosa et al., 2022). T7 RNAP (Steitz, 1998), T3 RNAP (Morris et al., 1986; Leary et al., 1991), and SP6 RNAP (Stump and Hall, 1993; Singh and Murali, 2023) are typical catalytic enzymes involved in RNA *in vitro* transcription. Among these, T7 RNAP, encoded by the bacteriophage T7, is the best-characterized member of a widespread family of RNAPs. T7 RNAP was successfully employed in the synthesis of BNT162b2, the first mRNA vaccine approved by the FDA (Vogel et al., 2021).

Unlike mRNA synthesis, linear RNA precursor cyclization is necessary for circRNA preparation *in vitro*. Several methods have been developed for *in vitro* ligation: chemical-based methods (Müller and Appel, 2017), enzyme methods (Abe et al., 2018; Kershaw and O'Keefe, 2012; Moore and Query, 2000), and ribozyme-based methods (Petkovic and Müller, 2013; Obi and Chen, 2021; Lee et al., 2022). The different methods for linear RNA precursor ligation have both advantages and limitations (Chen and Lu, 2021; Obi and Chen, 2021). Permuted introns and exons (PIE), which are ribozyme-based methods, are widely used for circRNA preparation (Puttaraju and Been, 1992; Wesselhoeft et al., 2018; Chen and Lu, 2021). Compared with chemical and enzyme-based methods showing decreased yields of circRNA products longer than 1 kb, the PIE method has wide adaptability to length (Obi and Chen, 2021). Previous research suggests that up to 5 kb of circRNA can be processed by the PIE method (Petkovic and Müller, 2015; Wesselhoeft et al., 2018).

**FIGURE 1:**
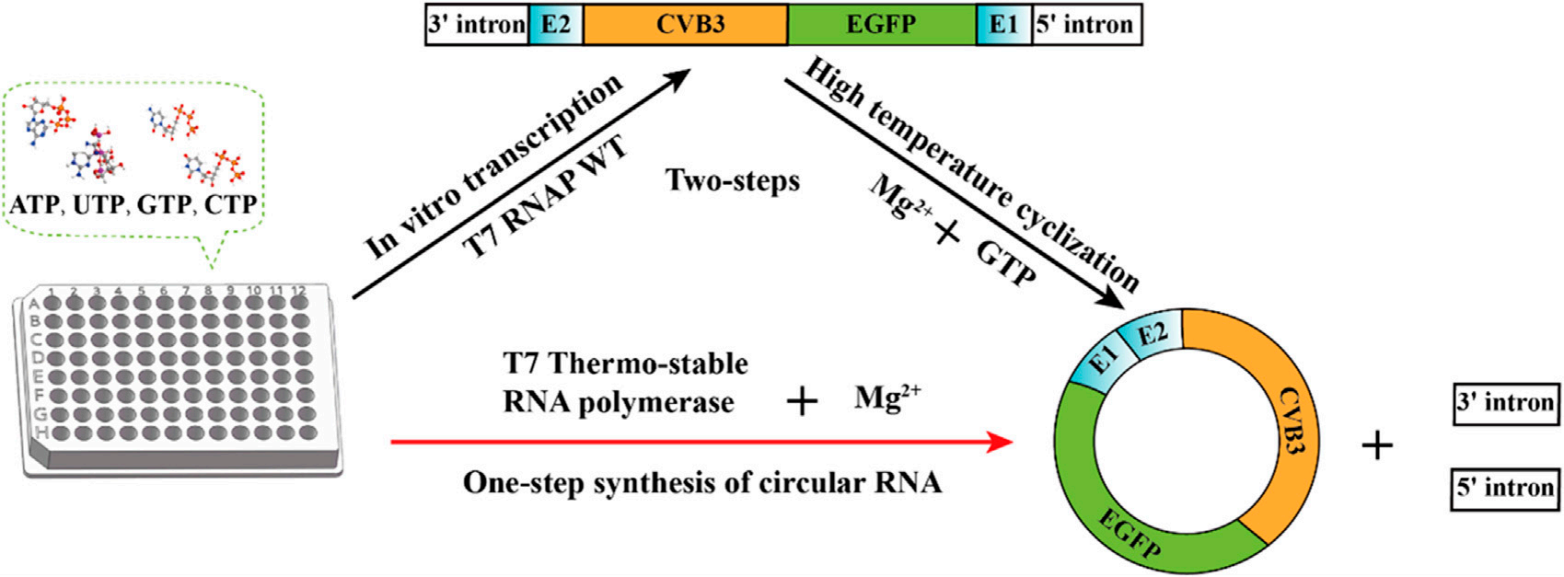
One-step synthesis of circular RNA.

The reaction conditions of the PIE method are relatively simple, with only GTP and Mg^2+^ added as cofactors, and the reaction temperature is much higher than that of *in vitro* transcription by T7 RNA polymerase (Wesselhoeft et al., 2018). Therefore, circRNA synthesis must be performed in two steps: linear RNA precursor synthesis and linear RNA precursor ligation. Our objective was to create a one-step reaction for circRNA synthesis using T7 RNAP as the catalyst. If the mutant thermostable T7 RNA polymerase is utilized, a single step of circRNA synthesis may be implemented (Wesselhoeft et al., 2018). In this study, we used consensus and folding free energy calculations for hotspot selection. Potential hotspots were selected after analysis. The specificity at high temperature, retained residue activity after high-temperature incubation, and values of *Tm* (melting temperature) were measured to confirm the improvement in T7 RNA polymerase thermostability. Subsequently, the thermostable T7 polymerase was efficiently used for the one-step synthesis of circular RNA (circRNA) (Figure 1).

## 2 Materials and methods

### 2.1 Strains, reagents, and chemicals

The pQE-30 plasmid and *Escherichia coli BL21(DE3)* were purchased from Novagen Inc. (Madison, WI, USA) for T7 RNAP heterologous expression. The site-directed mutagenesis kit and dsRNA quantitative kit were supplied by Vazyme Co., Ltd. (Nanjing, China). ATP, GTP, CTP, UTP, pyrophosphatase, and murine RNase inhibitor were purchased from Vazyme Co., Ltd. (Nanjing, China). MgCl_2_, glycerol, dithiothreitol (DTT), and other chemicals were purchased from Aladdin Industrial Inc. (Shanghai, China) and other commercial sources.

### 2.2 Plasmid design, cloning, and heterologous expression of T7 RNAP

The nucleotide sequence of T7 RNAP was obtained from GenBank NC_001604.1 and optimized using GeneOptimizer^®^ expert software for improved expression in *E. coli*. Optimized foreign nucleotide sequences have been targeted into plasmid pQE-30 digested with *BamHI* and *HindIII* (inserted His-tag sequences at 5ʹ terminal of foreign nucleotide sequences), which was then used for the transformation of *E. coli* BL21 (DE3) cells or as the original material for site-directed mutagenesis. The oligonucleotide primers required for site-specific mutations are detailed in Supplementary Table S1.

*E. coli* BL21 (DE3) harboring T7 RNAP-carrying recombinant pQE-30 was cultured in Luria-Bertani (LB) broth by supplementing 100 mM ampicillin at 37°C on a shaker (220 rpm). When the OD at 600 nm reached ∼0.6, 0.5 mM IPTG was added to the growing culture to induce T7 RNAP expression for 4–6 h at 37°C. Subsequently, cells were harvested by centrifugation (4°C, 10,000×*g*, 20 min). Thereafter, the cells were washed twice, resuspended in Tris-HCl buffer (pH 8.0, 50 mM pH 8.0), and disrupted by sonication (260 W, 3-s pulse, 5-s pause). Crude enzymes were obtained by centrifugation (4°C, 10,000×*g*, 20 min). The protein content was analyzed using SDS-PAGE.

### 2.3 Purification of T7 RNAP

Nucleic acid impurities were removed from the crude enzyme by pretreatment with the addition of 0.1 vol of 3% polyethyleneimine (PEI). The sample was diluted with the same volume of SP-binding buffer (20 mM MES, 100 mM NaCl, 5% v/v glycerol, 1 mM dithiothreitol, pH 6.5) and loaded onto a 1 mL SP column equilibrated with 10 mL SP-binding buffer. The sample was eluted with 5 mL SP elution buffer (40 mM MES, 500 mM NaCl, 5% v/v glycerol, 1 mM DTT, pH 7.5). Subsequently, T7 RNAP was diluted with the same volume of Ni-binding buffer (300 mM NaCl, 0.2 mM DTT, 20 mM imidazole, 5% v/v glycerol, 20 mM Tris-HCl pH 7.5) loaded onto a His trap HP (GE Healthcare). The purified T7 RNAP was eluted with 5 mL of Ni-elution buffer (200 mM NaCl, 0.2 mM DTT, 450 mM imidazole, 5% v/v glycerol, 20 mM Tris-HCl pH 7.5). Purified T7 RNAP was desalted using a HiTrap Desalting Column (GE Healthcare Corp., USA). The sample content was measured using a BCA Protein Assay Kit (Vazyme Co., Ltd., Nanjing, China) with bovine serum albumin (BSA) as a standard.

### 2.4 Measurement of specific activity

The activity of the samples (T7 RNAP wild type [WT] and mutants) was estimated as X U/μL. T7 RNAP standard (Vazyme Co., Ltd, Nanjing, China) and samples were diluted by dilution buffer (100 mM NaCl, 0.1 mM EDTA-2Na, 0.1% g/g Triton X-100, 10% v/v glycerol, 50 mM Tris-HCl pH 8.0) to 0 U/μL, 0.05 U/μL, 0.1 U/μL, 0.2 U/μL, 0.3 U/μL, and 0.6 U/μL. The reaction mixture (final concentrations of 0.5 mM ATP/GTP/CTP/UTP, 2 U/μL murine RNase inhibitor, 0.4% μg/μL DNA template, 20% v/v T7 RNAP, 1× transcription buffer pH 8.0) was added to a 96-well plate and incubated for 30 min at 37°C or other temperatures (the T7 RNAP standard was only incubated at 37°C). Subsequently, the nucleic acid dye BR reagent (Vazyme Co., Ltd., Nanjing, China) was added. Fluorescence detection was performed at an excitation wavelength of 630 nm, emission wavelength of 680 nm, and a gain value of 126. A linear regression equation was constructed using a microplate reader to determine slope and k. Specific activity was defined as follows:

$$Y\;\left(\text{Standard}\right)=k_{1}X+b,$$ (1)

$$Y\;\left(\text{Sample}\right)=k_{2}X+b,$$ (2)

$$\text{Activity of sample}=k_{1}/k_{2}\times \text{Estimated value of activity }\left(U/\mu L\right),$$ (3)

$$\text{Specific activity of sample}=\frac{\text{Activity}\;\text{of}\;\text{protein}\;\left(U/\;\mu L\;\right)}{\text{Concentration}\;\text{of}\;\text{protein}\;\left(\;\mu g/\mu L\right)}.$$ (4)

### 2.5 Analytical methods

T7 RNAP WT and variants were diluted by dilution buffer to 100 U/μL and pre-incubated at 45°C for different time intervals (0 min, 20 min, 40 min, and 60 min). Subsequently, the residue activity was measured using Eq. 4.

The melting temperature was measured as follows: T7 RNAP WT and 20 μL of variants (no less than 1 μg/μL in 200 mM NaCl, 0.2 mM DTT, 5%v/v glycerol, 0.1 mM EDTA-2Na, 50 mM Tris-HCl, pH 8.0) were added to 0.15 mL PCR tubes, and then measured by Prometheus Panta (NanoTemper, Germany) varying temperatures from 25°C to 95°C, with a heating speed of 1 °C/min.

### 2.6 One-step reaction for circRNA synthesis

The reaction mixture of circRNA synthesis (final concentrations of 7.5 mM ATP/GTP/CTP/UTP, 2 U/μL Murine RNase inhibitor, 0.005 U/μL pyrophosphatase, 2.5% μg/μL DNA template, 15 U/μL T7 RNAP, 1× transcription buffer (Vazyme Co., Ltd, Nanjing, China)) was added to a 96-well plate and incubated for 90 min at 37°C, 40°C, 45°C, 48°C, and 50°C. Subsequently, the RNA products were purified using clean RNA magnetic beads (Vazyme Co., Ltd., Nanjing, China). The RNA product yield was measured using a Nano-300 instrument. After denaturing the RNA products (incubated for 5 min at 70°C and then iced for 5 min at 0°C), the ratio of cyclization was measured by capillary electrophoresis (CE).

### 2.7 Measurement of dsRNA content

After circRNA synthesis with 20 mM Mg^2+^, 25 mM Mg^2+^, 30 mM Mg^2+^, 35 mM Mg^2+^, and 40 mM Mg^2+^ as the cofactor, the RNA product yield was measured using a Nano-300 instrument. dsRNA content was measured using a dsRNA quantitative kit supplied by Vazyme Co., Ltd (Nanjing, China). This kit uses a double-antibody sandwich enzyme-linked immunosorbent assay (ELISA) to detect dsRNA content in the *in vitro* transcription system.

## 3 Results and discussion

### 3.1 CircRNA synthesis using T7 RNAP WT

**FIGURE 2:**
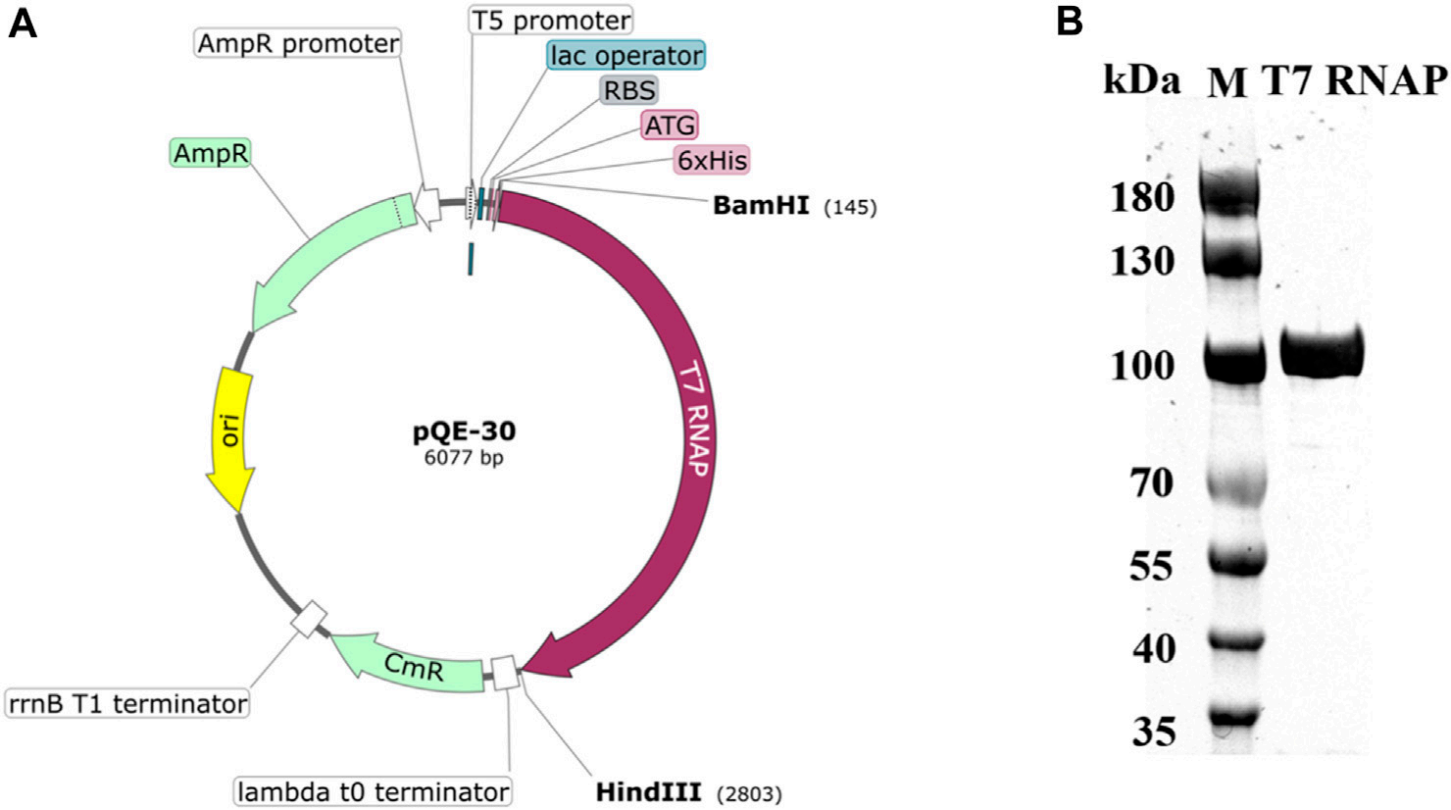
Structure of the T7 RNAP plasmid and SDS-PAGE analysis of His-tagged T7 RNAP. **(A)** Map of the recombinant plasmid pQE-30. **(B)** SDS-PAGE analysis of purified His-tagged T7 RNAP overexpressed in *Escherichia coli*.

The PIE method is an effective, easy, and widely used approach for RNA cyclization (Wesselhoeft et al., 2018; Chen and Lu, 2021). A one-step reaction for circRNA synthesis was attempted using T7 RNAP WT as the catalyst. Before catalysis, purified T7 RNAP WT was prepared. The optimized T7 RNAP WT gene, which was obtained from Bacteriophage T7, was amplified and overexpressed in *E. coli* BL21 (DE3). Additionally, the 6×His tag was expressed as an N-terminal fusion to the T7 RNAP. The encoded His-tagged T7 RNAP was purified as described in Section 2 and was obtained from the cell-free extract of the recombinant *E. coli* strain carrying the plasmid pQE-30 (Figure 2A). Subsequently, the purified His-tagged T7 RNAP produced a single band on SDS-PAGE with a molecular mass of approximately 100 kDa (Figure 2B).

The activity of T7 RNAP WT at different temperatures was then measured. The activity decreased rapidly as the temperature increased from 37°C to 50°C. The specificity activity of T7 RNAP WT at 45°C was only 22.09 ± 3.7 U/μg, which was more than 10 times less than the original specificity activity at 37°C. Thus, 37°C was an appropriate temperature for using T7 RNAP WT for RNA synthesis.

**FIGURE 3:**
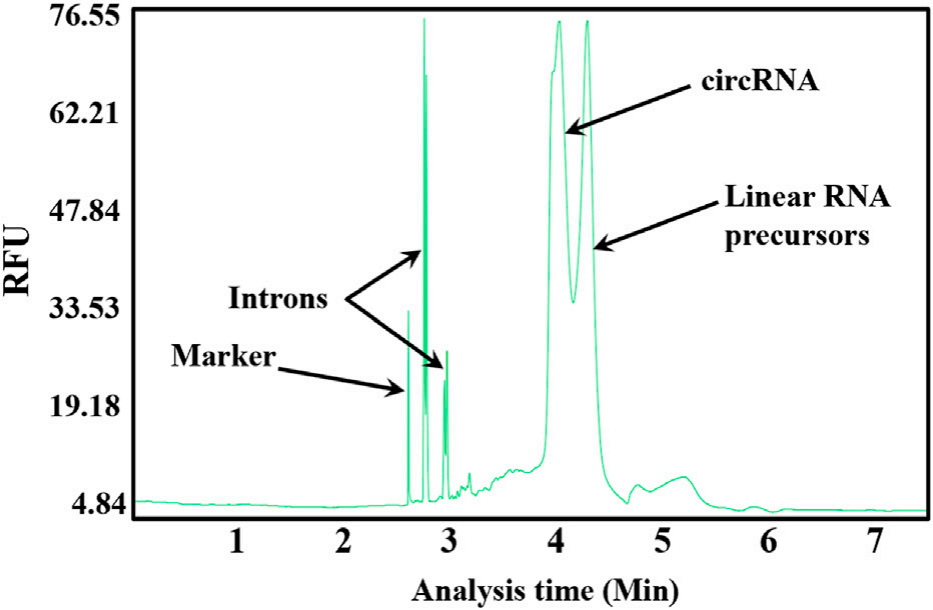
Capillary electrophoresis (CE) analysis of circRNA synthesis at the reaction temperature of 37°C.

Cofactors Mg^2+^ and GTP needed for cyclization are included in linear RNA precursor synthesis systems (Wesselhoeft et al., 2018). Thus, the one-step method for circRNA synthesis by T7 RNAP WT at 37°C was evaluated without adding any extra reagent. circRNAs were synthesized (Figure 3). However, nearly 50% of the linear RNA precursors were retained. The large amount of retained precursors is probably due to the low reaction temperature (Wesselhoeft et al., 2018). T7 RNAP WT is unstable at higher temperatures. Consequently, circRNA synthesis can be developed via a two-step approach for circRNA precursors by sequential conversion via transcription and cyclization. T7 RNAP thermostability modification should be designed for effective circRNA synthesis.

### 3.2 Optimization of T7 RNAP thermostability

#### 3.2.1 Selected substitutions increased specific activity

The thermostability modification of proteins is one of the most common directions of enzyme modification (Kellis et al., 1988; Pace et al., 2011; Watanabe et al., 2016; Ferenczy and Kellermayer, 2022; Elias et al., 2023). The thermostability of the T7 RNAP variants has been previously reported (Boulain et al., 2013; Meyer et al., 2015; Wu et al., 2020). Meyer et al. selected T7 RNAP mutants (S430P, N433T, S633P, F849I, and F880Y) and T7 RNAPs (S430P, N433T, S633P, F849I, F880Y, and P266L) as bases to further increase the activity of T7 RNA polymerases at higher temperatures and was successful (Meyer et al., 2015).

**FIGURE 4:**
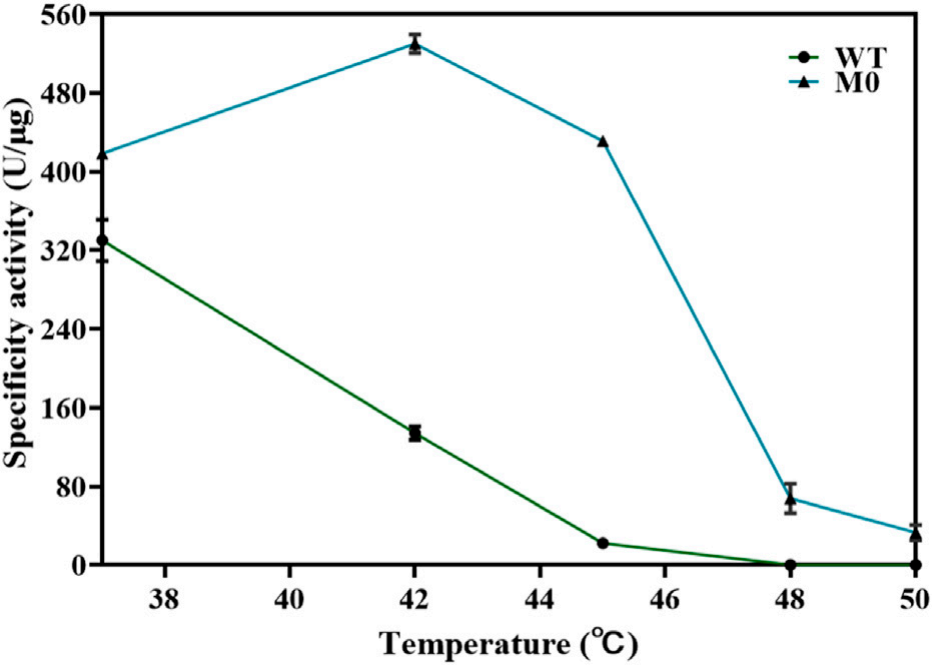
Thermostability of T7 RNAP M0 and WT at the reaction temperatures of 37°C–50 C.

T7 RNAP P266L might catalyze transcription by reducing short abortive products but slightly increasing extension products (Tang et al., 2014). The purpose of this work was to identify mutants as the basis for further research on T7 RNAP thermostability, and the application of P266L was not our priority in this work. Thus, T7 RNAPs (S430P, N433T, S633P, F849I, and F880Y) were selected as the original thermostable RNAP, M0. The activity of M0 was determined (Figure 4). Compared with the T7 RNAP WT, M0 showed significantly increased activity at high temperatures, which was consistent with previously reported results (Meyer et al., 2015). The specificity activity of M0 was 430.95 ± 5.15 U/μg at 45°C, which was more than 19-fold of T7 RNAP WT specificity activity. As the temperature increased, the M0 enzyme activity decreased sharply, and the enzyme activity declined to 67.78 ± 15.10 U/μg at 48°C. It was observed that M0 was almost deactivated at 50°C. The high-temperature tolerance of M0 was inadequate to better understand the effect of temperature on the cyclization rate. Binding to other hotspots is an appropriate method for improving thermostability (Boulain et al., 2013; Elias et al., 2023).

#### 3.2.2 Substitution screening via a thermostability prediction software program

Various prediction software programs have been used for hotspot selection, such as using I-Mutant/PoP MuSic/Deepddg to predict free energy calculations (Kwasigroch et al., 2002; Capriotti et al., 2005; Cao et al., 2019; Kumar et al., 2020), HoT Music for predicting changes in melting temperature (Dotsenko et al., 2023), and HotSpot Wizard/FireProt for integrating various computing tools for sequence consensus analysis, B-factor analysis, and free energy calculation (Bendl et al., 2016; Musil et al., 2017; Sumbalova et al., 2018). Multiple hotspots can be obtained through a single calculation; thus, the design of efficient thermal stability variants is simplified.

In this study, HotSpot Wizard, which integrates multiple computing tools, was chosen for T7 RNAP hotspot screening. The crystal structure of T7RNAP (Protein Data Bank code 1MSW) (Yin and Steitz, 2002) was used to compute the values of the free energy *N*-terminal domain and the partial C-terminal domain of T7 RNAP that underwent drastic changes in protein conformation from the initiation phase to the elongation phase (Yin and Steitz, 2002; Steitz, 2004; 2006; Borkotoky and Murali, 2018). No obvious change in conformation was observed between the palm domain and the C-terminal domain during transcription (including amino acid residues 412–553 and 785–879). Therefore, the palm domain was used as a hotspot-screening region to avoid conformational changes.

**FIGURE 5:**
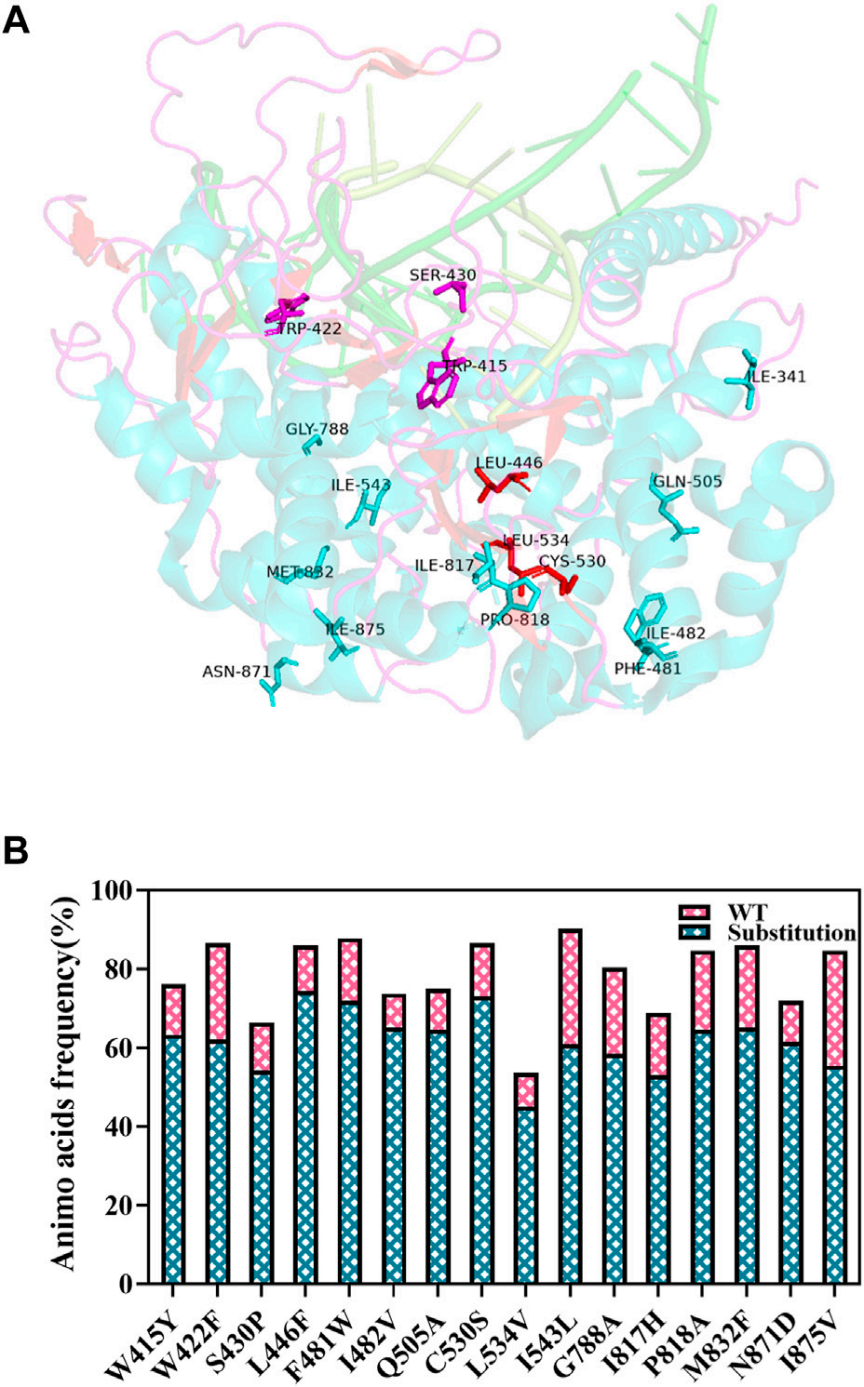
Position and frequency analysis of the set of mutations. **(A)** Position analysis of the set of mutations. **(B)** Potential substitutions screened by the frequency of amino acids.

The more amino acids that contribute to protein stability during evolution, the more conserved they become (Sumbalova et al., 2018). First, the amino acid consensus of the palm domain for initial hotspot screening by HotSpot Wizard was analyzed. Conserved amino acids may replace non-conserved amino acids to obtain more stable variants (Steipe, 1999; Porebski and Buckle, 2016). This method has been proven relatively reliable for improving protein thermostability (Wojcik et al., 2019; Hayashi et al., 2022; Michel et al., 2023). Sequences whose query identity was above 30% and below 90% are screened for the alignment of the T7RNAP homologs (Bendl et al., 2016). In addition, a 90% identity threshold of sequences was set in order to remove close homologs before the alignment (Bendl et al., 2016; Musil et al., 2017). Finally, 164 sequences were screened and aligned to estimate the conservation of each position in T7 RNAP (Supplementary Table S3). The rules of potential substitution selection were based on the frequency of occurrence: ≥50% of sequence homolog or at least ≥40% of analyzed sequences and ≥ 5-fold more than the frequency of the WT residue at the same time. The results of the consensus analysis are shown in Figures 5A, B (concatenated into one figure); a total of 16 potential hotspots were screened using the consensus analysis.

**FIGURE 6:**
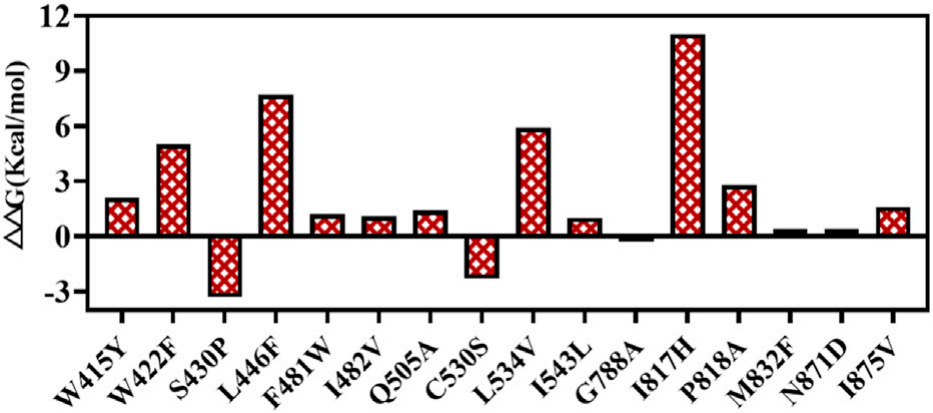
Values of ΔΔG.

A protein’s stability depends on its free energy. The folding free energies of the potential hotspots screened by Hotspot Wizard were evaluated. T7 RNAP S430P, C530S, and G788A had lower ΔΔG (<0 kcal/mol), which were −3.3, −2.3, and −0.3 kcal/mol, respectively (Figure 6). The 13 remaining potential hotspots had higher ΔΔG >0 kcal/mol. However, the substitution S430P had already been included in M0 (Meyer et al., 2015). Thus, T7 RNAP C530S and G788A were selected as potential hotspots.

### 3.3 Optimization of T7 RNAP thermostability variants

**FIGURE 7:**
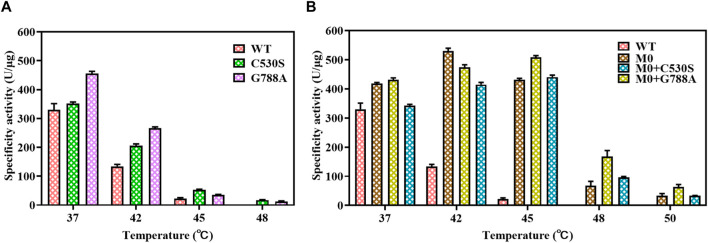
T7 RNAP variants improved specificity activity compared with T7 RNAP WT. **(A)** Single-site mutants improved specificity activity in high-temperature reactions. **(B)** Multisite mutants improved specificity activity in high-temperature reactions.

Protein expression was analyzed using SDS-PAGE (Supplementary Figure S2, Supplementary Material). There was no difficulty in the expression of T7 RNAP WT and mutants. The T7 RNAP specificity of the WT and its variants was further determined. As the data showed, the method described above was reliable for hotspot selection; both variants, G788A and C530S, improved protein activity by more than 50% at 42°C (Figure 7A). Based on further research, binding with M0 confirmed that the specificity increased with increasing temperature. Up to 45°C, the activity of T7 RNAP M0+G788A reached the highest (508.73 ± 5.40 U/μg), which was much higher than T7 RNAP WT at 37°C (Figure 7A; Figure 7B). In most cases, a loss of specificity activity was observed at 48°C. In particular, single-site mutants of T7 RNAP were inactivated. Only M0+G788A retained an activity of 168.08 ± 20.46 U/μg, whereas M0 and M0+C530S fell below 100 U/μg. M0+G788A resulted in optimized multiple hotspot-aggregated T7 RNAP variants, which retained 63.08 ± 8.92 U/μg when the temperature rose to 50°C. Obtaining better variants through multiple hotspot aggregations is an effective method for improving enzyme thermostability (Boulain et al., 2013; Elias et al., 2023).

It was observed that T7 TNAP M0+G788A performed much better than M0+C530S under higher-temperature conditions (≥48°C). To further understand T7 RNAP M0+G788A, its thermostability was measured under different time intervals of high-temperature incubation at 45°C. Under incubation conditions, T7 RNAP WT and G788A were inactivated after 20 min, whereas T7 RNAP M0+G788A retained more than 95% of its initial activity. After 60 min of incubation, T7 RNAP M0+G788A retained >300 U/μg activity. However, the basement-M0 dropped to 78.9 ± 5.88 U/μg, which was less than 20% of its initial activity. T7 RNAP M0+G788A still retained more than 50% of its initial activity after being incubated for 80 min at 45°C (Table 1). The results indicated that T7 RNAP M0+G788A, designed using the above strategy, was effective.

**TABLE 1:** Measurement of T7 RNAP variants and WT thermostability.

T7 RNAP	Specificity activity (U/μg)	Time (min)
WT	330.15 ± 21.15	0
M0	418.42 ± 3.69	0
G788A	455.3 ± 8.1	0
M0+G788A	431.52 ± 6.12	0
WT	—	20
M0	276.54 ± 15.64	20
G788A	—	20
M0+G788A	411.45 ± 19.48	20
M0	106.47 ± 9.53	40
M0+G788A	357.87 ± 18.21	40
M0	78.9 ± 5.88	60
M0+G788A	324.16 ± 7.92	60
M0	—	80
M0+G788A	274.22 ± 14.77	80
M0+G788A	168.38 ± 5.28	100

Measurement of the protein melting temperature (*Tm*) is another commonly accepted method for protein thermostability analysis (Senisterra and Finerty, 2009; Watanabe et al., 2016; Elias et al., 2023). The *Tm* of T7 RNAP was determined by dynamic light scattering (DLS), as previously reported (Prometheus Panta). As shown in Supplementary Figure S3, the *Tm* values of T7 RNAP WT and M0+G788A were estimated to be 39.91°C ± 0.03°C and 45.19°C ± 0.01°C, respectively. A thermostable variant was successfully constructed by combining M0 and G788A, achieving a high melting temperature that increased by 5°C.

Substitution with M0+G788A (S430P, N433T, S633P, F849I, F880Y, and G788A) significantly improved T7 RNAP stability at high temperatures. The optimized M0+G788A mutant showed higher enzyme activity than the WT. Furthermore, after incubation at 45°C for 80 min, 274.22 ± 14.77 U/μg of residue activity was retained. This variant was proficient in one-step circRNA synthesis.

### 3.4 Measurement of one-step circRNA formation

**FIGURE 8:**
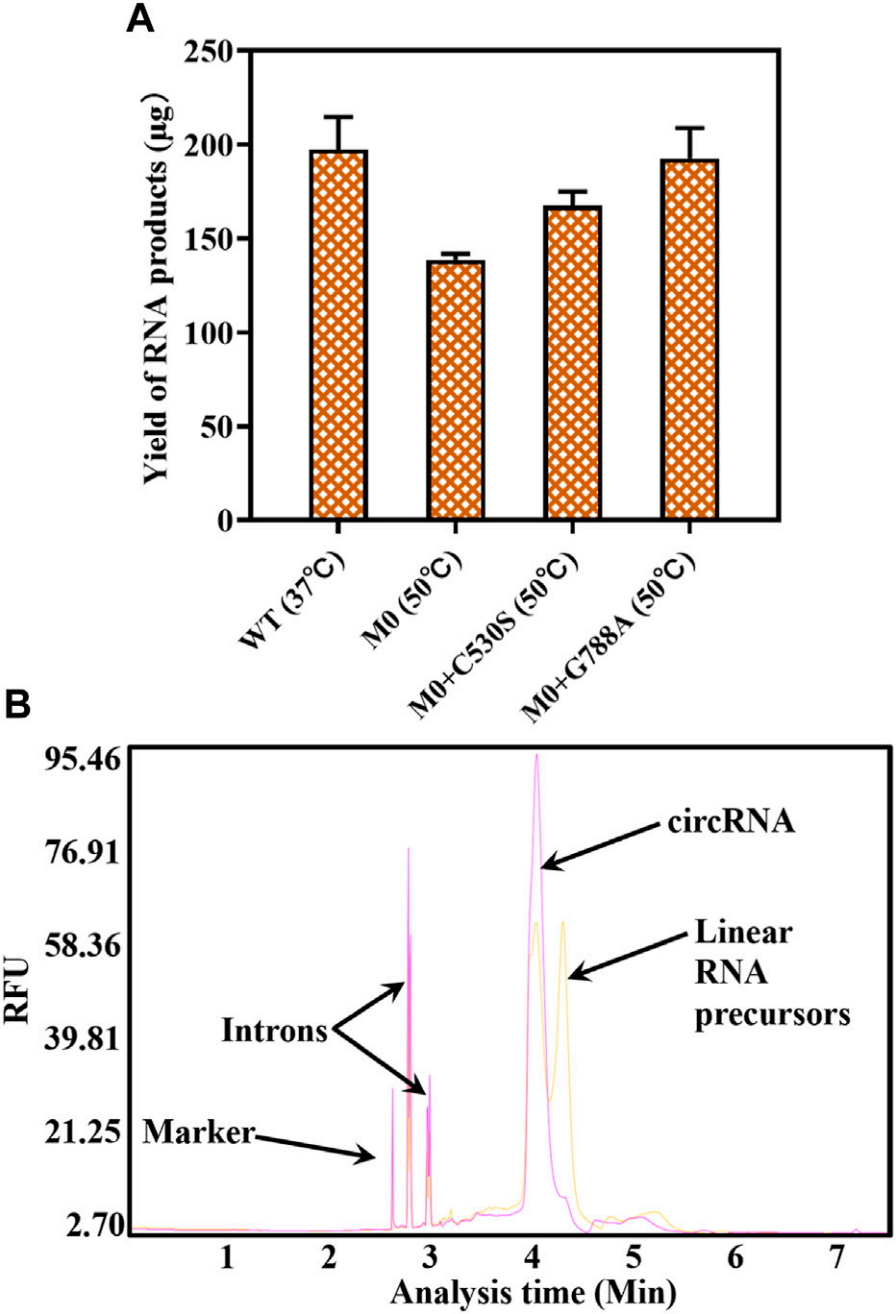
Yield of circRNA products and the product analysis by capillary electrophoresis (CE). **(A)** Yield of circRNA products through catalyzing by T7 RNAP WT or variants (WT 37°C/M0+G788A 50°C). **(B)** CE traces for circRNA production (yellow: circRNA synthesized using T7 RNAP WT as the catalyst at 37°C; red: circRNA synthesized using T7 RNAP M0+G788A as the catalyst at 50°C).

Before applying the T7 RNAP variant to one-step circRNA synthesis, different yields of RNA products were measured using T7 RNAP WT and its variants as catalysts. From the data shown in Figure 8A, by adding 300U of T7 RNAP WT to the reaction system, 197.3 ± 17.38 μg RNA products were acquired at the reaction temperature of 37°C after incubation for 90 min. As the reaction temperature rose to 50°C, 192.6 ± 16.2 μg circRNA products were achieved through 300U of M0+G788A as the catalyst. No significant decrease in the circRNA expression was observed. As we previously observed, WT was inactive at 50°C. When replaced by M0 as the catalyst, only 138.7 ± 3.13 μg circRNA products were measured (Figure 8A). Compared with M0, M0+C530S improved the yield of circRNA to 167.3 ± 7.67 μg at 50°C, which was still lower than using T7 RNAP WT as the catalyst at 37°C. Based on these findings, the problem of high-temperature intolerance of the catalyst used in circRNA synthesis was solved by constructing the T7 RNAP variant M0+G788A.

As measured in Section 3.1, nearly 50% of linear RNA precursors were retained in the one-step reaction for circRNA synthesis by T7 RNAP WT at 37°C (Figure 8B, yellow). The efficiency of T7 RNAP variant M0+G788A was measured when one-step circRNA synthesis was conducted at 50°C. As expected, linear RNA precursors were reduced to 4.5 ± 0.28% (Figure 8B Red, Supplementary Table S2). More than 95% of the linear precursors were cyclized when transcribed without any additional reagents.

**FIGURE 9:**
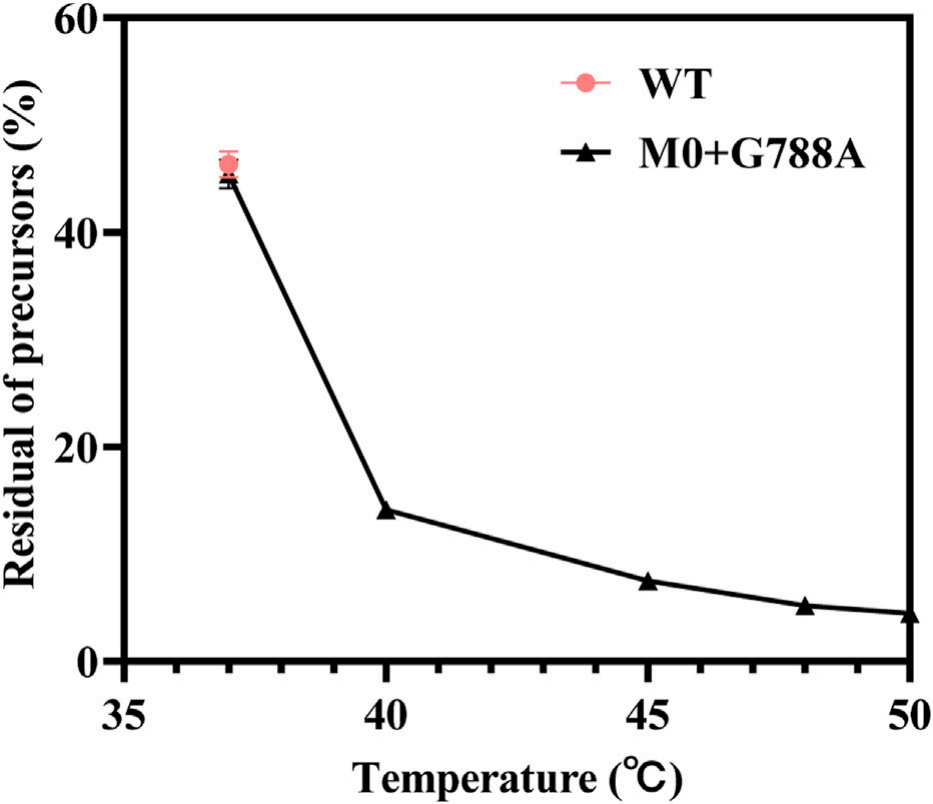
Minor residual content of precursors retained during high-temperature vitro transcription reactions at the temperature 37°C–50 C [red: T7 RNAP WT as the catalyst (37°C); black: T7 RNAP M0+G788A as the catalyst (37°C–50 C)].

To understand the relationship between temperature and cyclization ratio, one-step circRNA cyclization reactions were conducted at different temperatures. As shown in Figure 8B, the ratio of cyclization gradually increased as the temperature increased from 37°C to 50°C. The precursor residue decreased sharply as temperature increased. As the temperature rose to ≥45°C, the precursor residue retained was ≤7.5 ± 0.14%, which was less than 10% of total RNA products. When the temperature rose to 50°C, the precursor residue only retained 4.5 ± 0.28% of total RNA products (Figure 9, Supplementary Table S2). Precursors decreased by more than 90% compared to the precursors retained in the low-temperature (37°C) reaction.

Wesselhoeft et al. performed high-temperature incubation to form rings and required step-by-step addition and incubation to obtain high-cyclization-rate IVT products (Wesselhoeft et al., 2018). The T7 RNAP M0+G788A variant constructed in this study successfully catalyzed a high cyclization ratio of circRNA products at high temperatures without any additional reagents. CircRNA formation in only one step was proven to be successful.

### 3.5 DsRNA byproducts were reduced in high-temperature transcription

In addition to the simplified synthesis process, circRNA synthesis also faces the common problems of *in vitro* transcription. Various byproducts are produced during product-templated transcription (Donghi and Schnabl, 2011; Wu et al., 2020; Dousis et al., 2023). Among them, the dsRNA byproduct, which may result in a strong immune response against RNA drugs, has been detected by researchers (Kato et al., 2006; Pichlmair et al., 2006; Goubau et al., 2014). dsRNA byproducts were produced at a proportion of 1.98 ± 0.04 ×10^–4^ of the total yield of RNA products catalyzed by T7 RNAP WT at 37°C (Supplementary Table S2). Although their content was relatively low, the strong immune response caused by dsRNAs cannot be ignored (Wu et al., 2020; Dousis et al., 2023).

There are several features that drive the production of dsRNA byproducts, such as the 3′-extension of RNA transcribed *in vitro*, the production of antisense RNAs and so forth (Cavac et al., 2021; Dousis et al., 2023). In a previous study, modified ribonucleotides were shown to reduce the synthesis of antisense RNA, which might induce dsRNA production (Nance and Meier, 2021). Karikó et al. used modified ribonucleotides to reduce the production of dsRNA and improve the efficiency of mRNA translation (Karikó and Weissman, 2007). Because of this innovation, she won the 2023 Nobel Prize for Physiology and Medicine. When modified ribonucleotides are used for circRNA synthesis, the splicing efficiency is dramatically reduced, which induces the relatively low rate of cyclization (Wesselhoeft et al., 2019). Thus, this method has proven to be unsuccessful for dsRNA-reduced circRNA synthesis.

**FIGURE 10:**
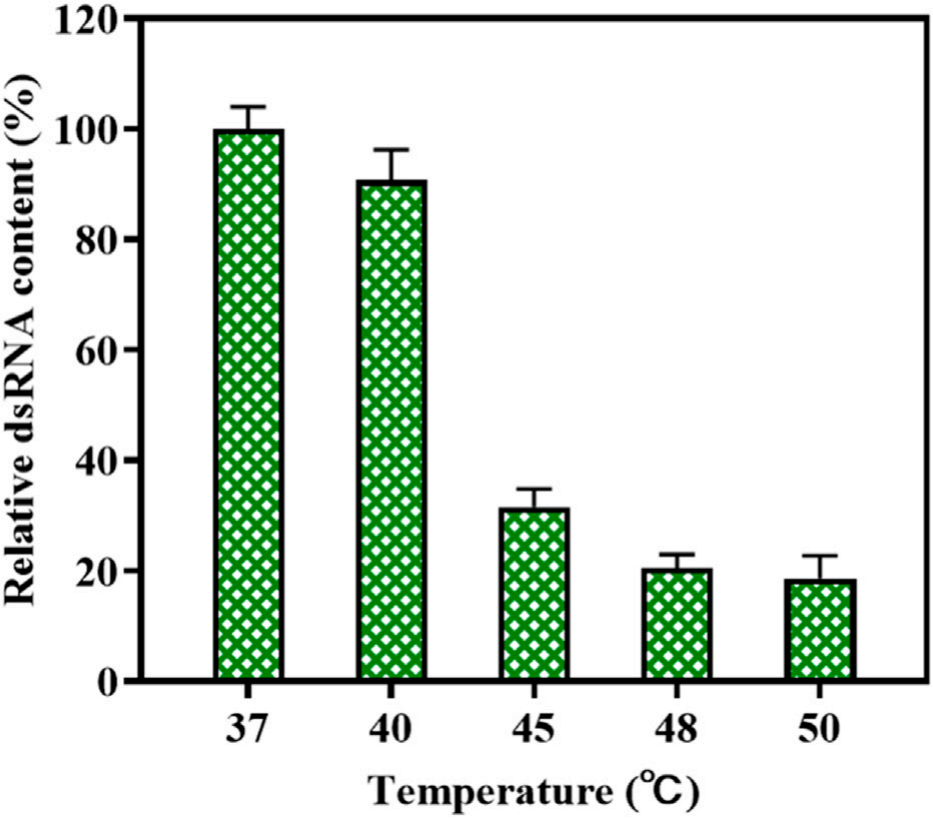
Reduction of relative dsRNA byproduct production during the high-temperature vitro transcription reaction (37°C–50 C).

Wu et al. found that the high-temperature transcription reaction could reduce the production of 3′-extension products of mRNA (Wu et al., 2020). This method may also apply to the dsRNA-mediated synthesis of circular RNA. We measured the amount of dsRNA produced by high-temperature transcription catalyzed by the T7 RNAP variant M0 + G788A. The rate of dsRNA production via T7 RNAP WT as the catalyst was plotted at 100. As shown in Figure 10, the content of dsRNA byproducts decreased rapidly as the reaction temperature rose. As the temperature rose to 45°C, relative dsRNA content decreased to 31.54% ± 3.21% of its initial level. When the temperature was further raised to 50°C, only 18.6% ± 4.13% of dsRNA byproducts were produced in *in vitro* transcription compared to the production of dsRNA in the reaction at 37°C. Thus, the application of T7 RNAP M0+G788A in high-temperature circRNA synthesis not only simplified the synthesis process but also reduced dsRNA production. However, there are still some limitations via T7 RNAP M0+G788A as the catalyst for dsRNA decrease. Transcription in the high-temperature vitro reaction cannot decrease the production of antisense RNAs, which is an important feature that drives the production of dsRNA byproducts (Wu et al., 2020; Nance and Meier, 2021).

## 4 Conclusion

In this study, we aimed to optimize a one-step synthesis of circRNA (circRNAs). A high temperature was found to be a crucial factor in achieving a high ratio of cyclization products. However, T7 RNAP WT was unstable at high temperatures. Therefore, it was necessary to study thermostable RNA polymerases. Effective hotspots were optimized through methods such as the analysis of amino acid consensus and computing the values of the free energy of T7 RNAP. Due to further screening and superposition, the multiple hotspot aggregation of the thermostable T7 RNAP variant M0+G788A (S430P, N433T, S633P, F849I, F880Y, and G788A) was optimized for simplified circRNA synthesis. As expected, the cyclization rate increased with increasing temperature. When the temperature rose to 50°C, the cyclization rate exceeded 95%. The dsRNA byproduct, which could cause significant immunogenicity, decreased to less than 20% of its initial level at the same time, owing to the high temperature of the IVT reaction. Overall, this study contributes to the development of novel circRNA synthesis methods. Application of T7 RNAP M0+G788A for one-step circRNA synthesis was successfully demonstrated.

## Data Availability

The datasets presented in this study can be found in online repositories. The names of the repository/repositories and accession number(s) can be found in the article/Supplementary Material.
