# Supplementary material for: Effective synthesis of circRNA via a thermostable T7 RNA polymerase variant as the catalyst
Source: Front Bioeng Biotechnol. 2024 Apr 9;12:1356354. doi: 10.3389/fbioe.2024.1356354 (PMC11035883; doi:10.3389/fbioe.2024.1356354)
Supplement: Supplementary file 1 [file DataSheet2.docx]

Effective synthesis of circRNA via a thermostability T7 RNA polymerase variant as the catalyst

Table of Contents

Agarose gel electrophoresis of the DNA of T7 RNAP 3

SDS-PAGE analysis of T7 WT and thermos-stable variants 3

Values of melting temperature (*Tm*) 4

Primers for gene amplification 4

Proportion of each RNA species 5

T7 RNAP animo acids sequences used for consensus alignment 5

DNA and amino acids sequence of T7 RNAP 16


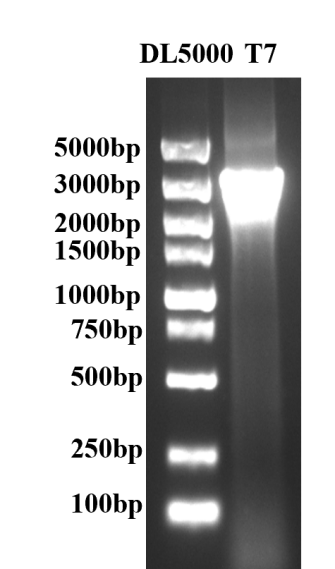


**Figure S1**. Agarose gel electrophoresis of the DNA of T7 RNAP


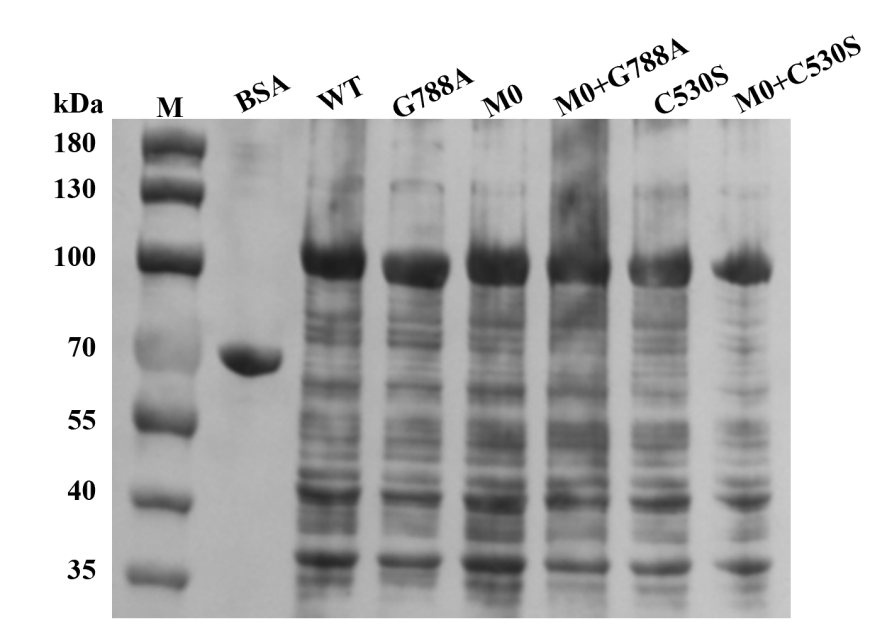


**Figure S2**. SDS-PAGE analysis of T7 WT and thermos-stable variants


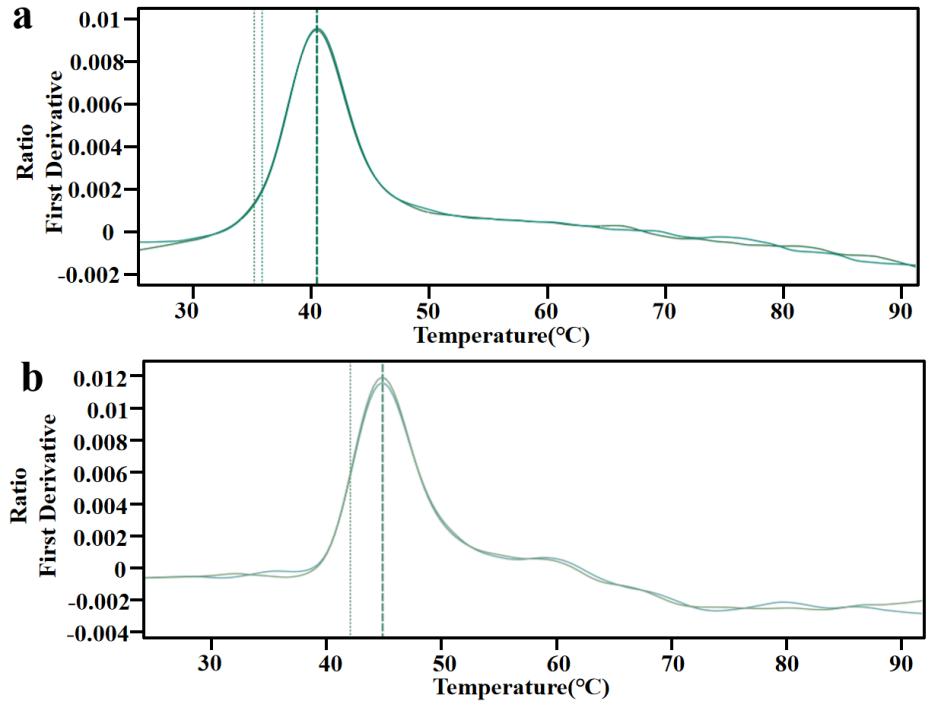


**Figure S3**. Values of melting temperature (*Tm*)

a: *Tm* values of T7 RNAP WT；b: *Tm* values of T7 RNAP M0+G788A

**Table S1.** Primers for gene amplification

| Primers | Sequence (5’-3’) |
| --- | --- |
| M0-S430P-N433T-F | GTAccgatgttcaccCCGCAGGGTAATGACATGACC |
| M0-S430P-N433T-R | CGGggtgaacatcggTACCGCGTAAACACGACCACG |
| M0-S633P-F | CAAGCGTccgGTGATGACCCTTGCTTACGGCA |
| M0-S633P-R | TCATCACcggACGCTTGGTCACGGAGCGAGTA |
| M0-F849I-F | TGATCAGataGCCGACCAACTGCATGAGTCCC |
| M0-F849I-R | GGTCGGCtatCTGATCATAAAAGTCTGCCAGAACA |
| M0-F880Y-F | ATCTGATtacGCCTTCGCGTAAAAGCTTAATT |
| M0-F880Y-R | CGAAGGCgtaATCAGATTCCAGGATGTCGCGG |
| M0+C530S-F | CTATAACagcAGCCTGCCGCTGGCCTTTGATG |
| M0+C530S-R | GCAGGCTgctGTTATAGCTCAGGCCGTGGTGC |
| M0+G788A-F | TCACTCACAGGACgctTCCCATCTGCGTAAGACCGT |
| M0+G788A-R | AagcGTCCTGTGAGTGAACGAAATTGGGTGCG |

**Table S2.** Proportion of each RNA species

| Enzyme | Linear precursors retained (%) | Linear precursors  Cyclized (%) | dsRNA (%) |
| --- | --- | --- | --- |
| WT (37℃) | 46.35±1.20 | 57.32±1.07 | (1.98±0.04) ×10^-2^ |
| M0+G788A (37℃) | 45.45±1.34 | 54.57±1.27 | (2.49±0.10) ×10^-2^ |
| M0+G788A (40℃) | 14.15±0.49 | 85.87±0.48 | (2.26±0.14) ×10^-2^ |
| M0+G788A (45℃) | 7.50±0.14 | 92.52±0.12 | (0.79±0.08) ×10^-2^ |
| M0+G788A (48℃) | 5.20±0.14 | 94.83±0.18 | (0.51±0.06) ×10^-2^ |
| M0+G788A (50℃) | 4.5±0.28 | 95.50±0.31 | (0.46±0.10) ×10^-2^ |

Table S3. T7 RNAP animo acids sequences used for consensus alignment

| number | Species name | Accession No. | Number | Species name | Accession No. | Number | Species name | Accession No. |
| --- | --- | --- | --- | --- | --- | --- | --- | --- |
| 1 | Enterobacteria phage T7 | / | 56 | *Nitrospira* sp. SG-bin2 | A0A1W9GIB5 | 111 | *Ralstonia* phage RSB1 | B5BTW2 |
| 2 | *Stenotrophomonas* phage IME15 | K4NXC1 | 57 | *Rhizobium radiobacter* ATCC 4718 | A0A1B9UVK7 | 112 | *Escherichia* phage K1E | Q2WC24 |
| 3 | Enterobacteria phage T3 | P07659 | 58 | *Methylovorus* sp. MM2 | A0A1A9T4G1 | 113 | *Brevundimonas* sp. GW460-12-10-14-LB2 | A0A160HX60 |
| 4 | *Yersinia* phage vB_YenP_AP10 | A0A0P0M6B3 | 59 | *Stenotrophomon-as* maltophilia SKK35 | M5CVP8 | 114 | Sphingomonas phyllosphaerae DSM 17258 | UPI0004030E40 |
| 5 | Enterobacter phage phiEap-1 | A0A0K2FHD8 | 60 | *Mesorhizobium* sp. LNJC384A00 | UPI0003CF5DAE | 115 | *Aminobacter* sp. J15 | UPI000463D846 |
| 6 | *Yersinia* phage Yepe2 | B3VCH9 | 61 | Mesorhizobium sp. WSM4312 | UPI000BB03EA1 | 116 | *Ralstonia* phage RSJ2 | A0A068Q5R3 |
| 7 | Enterobacteria phage K11 | P18147 | 62 | *Rhizobium* sp. CF097 | UPI00068B9CCE | 117 | *Ralstonia* phage RSJ5 | A0A077KYK0 |
| 8 | *Yersinia* phage fPS-21 | A0A2D0PE32 | 63 | Rhizobium loti DSM 2626 | UPI000C7C9039 | 118 | *Xanthomonas* phage phiL7 | C4ML35 |
| 9 | *Morganella* phage MmP1 | D1FNQ5 | 64 | Brucella pituitosa DSM 22207 | UPI0009A1F060 | 119 | *Sphingobium bisphenolivorans* DSM 102172 | UPI00039B03BF |
| 10 | *Erwinia* phage vB_EamP-L1 | G0YQ47 | 65 | Nitrincola sp. A-D6 | A0A099PAW9 | 120 | Rhizobiales bacterium TMED227 | A0A1Z9VR06 |
| 11 | *Pectobacterium* phage PP81 | A0A1L7DRY0 | 66 | *Mesorhizobium* phage vB_MloP_Lo5R7ANS | A0A076YJ28 | 121 | *Marinobacter* sp.(unknown) | A0A2D8DUN9 |
| 12 | *Pectobacterium* phage DU_PP_II | A0A2D2W5U8 | 67 | *Desulfovibrio* sp. 6_1_46AFAA | G1UXD8 | 122 | *Brevundimonas* sp. KM4 | A0A0F3L7W0 |
| 13 | *Escherichia* phage K1F | Q3YJZ7 | 68 | *Candidatus Pelagibacter* sp | A0A2G2ABF6 | 123 | *Pectobacterium* phage PP1 | I7FXR7 |
| 14 | *Citrobacter* phage CR8 | W6PP41 | 69 | *Rhizobium fredii* PCH1 | A0A2A6LSV5 | 124 | *Xanthomonas arboricola* ATCC 49083 | UPI0009B9AB36 |
| 15 | *Vibrio* phage ICP3 | F1D002 | 70 | *Candidatus Endolissoclinum* sp. TMED37 | A0A1Z8SNV0 | 125 | *Salinivibrio kushneri* CECT 9177 | UPI000988A2CE |
| 16 | *Pseudomonas* sp. SID14000 | UPI000B3C28C7 | 71 | *Desulfovibrio* sp. MES5 | A0A231P0U9 | 126 | *Vibrio parahaemolyticus* DSM 10027 | UPI00079FE2AE |
| 17 | *Pseudomonas* phage Pf-10 | A0A0A0YSI2 | 72 | *Phenylobacterium* sp. RIFCSPHIGHO2_01_FULL_70_10 | A0A1G2WI53 | 127 | uncultured *Alphaproteobacteria* bacterium | A0A1B0Z1L8 |
| 18 | *Pseudomonas* phage UNO-SLW3 | A0A1B2AMZ7 | 73 | *Stenotrophomon-as maltophilia* | A0A209LG30 | 128 | *Pseudomonas monteilii* DSM 14164 | A0A136Q9S6 |
| 19 | *Pseudomonas* phage phiIBB-PF7A | E9KIE1 | 74 | Ensifer aridi HAMBI 3707 | UPI000A1226FB | 129 | Candidatus Pelagibacter sp. TMED165 | A0A1Z9IUA9 |
| 20 | *Pseudomonas* phage shl2 | A0A160SY77 | 75 | *Inquilinus limosus* DSM 16000 | UPI000416AE03 | 130 | uncultured phage_Deep1-GF2-KM23-C739 | A0A1B1IVX7 |
| 21 | *Pseudomonas fuscovaginae* DSM 7231 | UPI0006B42879 | 76 | *Verrucomicrobia bacterium* TMED44 | A0A1Z8STP7 | 131 | *Alphaproteobacteria* | UPI0009E9629B |
| 22 | *Pseudomonas* phage phi15 | F0V6X0 | 77 | *Burkholderia* | UPI000B79BD2A | 132 | *Phaeodactylum tricornutum* (CCAP 1055/1) | B7GAR6 |
| 23 | Pseudomonas sp. MPBC4-3 | UPI000C88CD09 | 78 | *Vibrio* phage phi-A318 | H6WCV4 | 133 | *Alteromonas* sp. (unknown) | A0A2D7ESI3 |
| 24 | *Pseudomonas* phage gh-1 | Q859H5 | 79 | *Dialister micraerophilus* UPII 345-E | E4LAB3 | 134 | uncultured phage_Deep-GF0-KM16-C193 | A0A1B1IWU5 |
| 25 | *Pseudomonas* phage PPpW-4 | V5YUU1 | 80 | *Lysobacter capsici* AZ78 | A0A120AFY3 | 135 | *Blastocystis hominis Singapore isolate B* | D8LUV8 |
| 26 | *Pseudomonas* sp. B14 (2017) | UPI000A1E3D53 | 81 | *Ralstonia* phage RS-PI-1 | A0A1S6L1D6 | 136 | *Anthurium amnicola*(unknown) | A0A1D1Z5L1 |
| 27 | *Raoultella ornithinolytica* DSM 7464 | UPI000C290D86 | 82 | *Vibrio panuliri* DSM 27724 | UPI000952C807 | 137 | *Medicago truncatula* A17 | G7KS02 |
| 28 | *Pasteurella* phage vB_PmuP_PHB02 | A0A1Y0SYF0 | 83 | *Paraburkholderi-a usongensis* LMG 2954 | A0A1X7KQZ2 | 138 | *Acanthamoeba castellanii* ATCC 3001 | Q0QFR1 |
| 29 | *Pseudomonas fildesensis* DSM 102036 | A0A0J8FQB0 | 84 | *Rhodobiaceae bacterium* MED766 | A0A2E1QP68 | 139 | *Achromobacter* sp. DH1f | UPI0004682345 |
| 30 | *Ralstonia* phage RSB2 | E5RUZ8 | 85 | *Burkholderia* phage Bp-AMP4 | A0A0A8KWP2 | 140 | *Ralstonia solanacearum* DSM 9544 | A0A177RMK7 |
| 31 | *Delftia* phage IME-DE1 | A0A0F7INH1 | 86 | *Xylella* phage Paz | V5Q7M3 | 141 | *Liceales* sp. orange ball | Q0QFS3 |
| 32 | *Ralstonia* phage phiITL-1 | A0A0U1ZDN8 | 87 | *Caulobacter* phage Percy | A0A0M4RSN8 | 142 | *Populus trichocarpa* cv. Nisqually | U5GL15 |
| 33 | *Ralstonia* phage DU_RP_I | A0A2D2W578 | 88 | *Burkholderia ubonensis* DSM 17311 | A0A108BJB5 | 143 | *Kluyveromyces dobzhanskii* CBS 2104 | A0A0A8L473 |
| 34 | *Pseudomonas putida* DSM 6125 | Q88KM4 | 89 | *Burkholderia* sp. MSMB0265 | A0A1Y9SS22 | 144 | *Pseudomonas fildesensis* DSM 102036 | A0A0J8FQ91 |
| 35 | *Yersinia kristensenii* CFSAN060539 | A0A209AR39 | 90 | *Escherichia* phage ECBP5 | A0A0F6N5K2 | 145 | *RhodoPseudomonas palustris* ATCC BAA-98 | Q6N6T7 |
| 36 | *Polynucleobacter* sp. 39-45-136 | A0A259PLH2 | 91 | *Ralstonia solanacearum* DSM 9544 | A0A177S382 | 146 | *Marinobacter similis* JCM 19398 | W5YMV5 |
| 37 | *Chromobacterium haemolyticum* H4137 | A0A1W0DCV4 | 92 | *Burkholderia* sp. BDU5 | UPI00075D63BB | 147 | *Emiliania huxleyi* NIES-837 | R1CSH1 |
| 38 | *Devosia* sp. 66-22 | A0A1M3KT00 | 93 | *Agrobacterium* phage Atu_ph02 | A0A223VZI2 | 148 | *Heterolobosea* sp. OSA | Q0QFR9 |
| 39 | *Neorhizobium galegae* bv. orientalis str. HAMBI 540 | A0A068SMS8 | 94 | *Sphingopyxis* flava R11H | A0A1T5BP89 | 149 | *Arundo donax* (unknown) | A0A0A9V3U9 |
| 40 | *Chromobacterium violaceum* DSM 30191 | UPI0009D9FD77 | 95 | Enterobacteria phage SP6 | P06221 | 150 | *Ralstonia* phage phiITL-1 | A0A0U1ZE07 |
| 41 | *Propionispora hippei* DSM 15287 | A0A1M6MEA8 | 96 | Blood disease bacterium R229 | G2ZVZ6 | 151 | *Asaia prunellae* DSM 23028 | UPI00046EE0B8 |
| 42 | *Rhizobium radiobacter* DSM 9674 | A0A083ZR97 | 97 | *Pelagibacter* phage HTVC019P | M1ID95 | 152 | *Sesamum indicum* cv. 4294 | UPI0009D6A335 |
| 43 | unclassified *Thioalkalivibrio* | UPI0003612961 | 98 | *Methylophilales bacterium* 28-44-11 | A0A258Q2S4 | 153 | *Sporidiobolus salmonicolor* ATCC 36400 | E0XNZ6 |
| 44 | *Desulfovibrio oxyclinae* DSM 11498 | UPI00036A744C | 99 | *Pseudomonas* phage uligo (unknown) | A0A2H4P7Q2 | 154 | *Bursaphelenchus xylophilus* | A0A1I7SPB9 |
| 45 | *Endozoicomonas ascidiicola DSM 100913* | UPI00082D3025 | 100 | *Rhizobacter* sp. Root1221 | A0A0Q6XXA9 | 155 | *Ralstonia solanacearum* DSM 9544 | A0A0S4XK53 |
| 46 | *Rhizobium mongolense* subsp. *loessense* CCBAU 7190B | A0A1G4T8V0 | 101 | *Campylobacter fetus* subsp. *fetus* 006A-0059 | A0A0S4RDQ4 | 156 | *Halomonas beimenensis B*CRC 17999 | A0A291P582 |
| 47 | *Rhizobium* phage RHEph01 | L7TQW5 | 102 | *Burkholderia humptydooensis*(unknown) ATCC BAA-2767 | A0A0D5LFP9 | 157 | *Vibrio* phage 1032 | C3VVU3 |
| 48 | *Chelatococcus sambhunathii* DSM 18167 | A0A0K6HJC9 | 103 | *Proteus* phage PM 85 | A0A0F6NYB6 | 158 | *Stegodyphus mimosarum*(unknown) | A0A087V0W0 |
| 49 | *Rhizobium radiobacter* NRRL B-11291 | A0A0X8J309 | 104 | *Burkholderia ubonensis* DSM 17311 | UPI0008FDA309 | 159 | *Brassica campestris*(unknown) | UPI0008727CF8 |
| 50 | *esorhizobium* sp. LNJC405B00 | X5Y418 | 105 | *Acinetobacter* sp. DSM 17874 | A0A2D7VGV1 | 160 | *Nicotiana sylvestris*(unknown) | A0A1U7XSL2 |
| 51 | *Lentimicrobiaceae bacterium* | A0A2E2Y017 | 106 | *Burkholderia ubonensis* DSM 17311 | UPI0007567617 | 161 | *Brassica napus(*unknown) | A0A078JZ27 |
| 52 | *Methylobacterium* sp. ZNC0032 | UPI0006462269 | 107 | *Novosphingobium lindaniclasticum* LE124 | T0IL79 | 162 | *Vitis vinifera(u*nknown) | A5AW22 |
| 53 | *Stutzerimonas stutzeri* DSM 50227 | UPI0002E51E07 | 108 | *Vibrio* phage VEN | A0A2H5BMU1 | 163 | *Nicotiana sylvestris*(unknown) | A0A1U7Y1E7 |
| 54 | *Azorhizobium caulinodans* DSM 5975 | A8IFE5 | 109 | *Brevundimonas nasdae* TPW30 | A0A0B4CF06 | 164 | *Oceanospirillaceae bacteriumk* (unknown) | A0A2E9DCI2 |
| 55 | *Luteibacter s*p. 22Crub2.1 | A0A1T5BSF2 | 110 | *Burkholderia ubonensis* MSMB1754 | A0A104JDG1 |  |  |  |

DNA and amino acids sequence of T7 RNAP

Optimized cDNA sequence:

ATGAATACAATTAACATAGCTAAAAATGATTTCAGCGATATTGAACTGGCTGCGATCCCGTTTAATACCTTGGCGGACCATTATGGTGAGCGTCTGGCGCGTGAGCAACTTGCTCTGGAGCACGAATCCTACGAGATGGGTGAAGCGCGTTTCCGGAAGATGTTCGAGCGCCAGCTTAAGGCTGGCGAGGTGGCGGATAACGCTGCTGCGAAGCCGTTGATTACGACCCTGCTGCCGAAAATGATTGCGCGTATTAACGATTGGTTCGAGGAAGTGAAAGCAAAGCGTGGTAAACGTCCAACCGCATTCCAGTTTCTGCAAGAGATCAAGCCGGAGGCGGTGGCATACATTACGATCAAAACCACTCTCGCATGTCTCACCAGCGCGGATAATACTACGGTCCAAGCGGTGGCCAGCGCGATTGGCCGCGCGATTGAGGACGAAGCGCGTTTTGGTCGCATTCGTGACCTGGAAGCGAAACATTTTAAGAAGAACGTTGAAGAACAATTGAATAAACGCGTGGGCCACGTTTACAAGAAAGCGTTCATGCAAGTCGTTGAAGCTGATATGCTGAGCAAAGGCCTGTTAGGTGGTGAAGCATGGAGCAGCTGGCATAAGGAGGACTCAATTCACGTCGGCGTGCGGTGCATTGAAATGCTGATCGAATCGACCGGTATGGTTAGCCTGCATCGCCAAAACGCCGGCGTGGTGGGTCAAGATAGCGAAACGATTGAACTGGCGCCAGAGTACGCGGAAGCTATCGCTACCAGAGCCGGCGCATTGGCGGGGATCAGCCCGATGTTCCAGCCGTGTGTTGTACCACCGAAGCCGTGGACCGGTATCACCGGTGGAGGTTACTGGGCGAATGGTAGACGTCCGTTGGCGTTGGTTCGTACCCATAGCAAAAAGGCTCTGATGCGTTATGAAGACGTTTACATGCCGGAAGTGTACAAAGCCATCAACATCGCGCAGAACACTGCGTGGAAAATCAACAAAAAGGTGTTAGCTGTGGCGAACGTAATTACAAAATGGAAGCACTGCCCGGTTGAGGACATCCCGGCTATTGAACGTGAAGAGCTGCCAATGAAACCAGAGGACATTGACATGAATCCGGAGGCGCTGACCGCATGGAAACGTGCGGCAGCAGCGGTCTATCGTAAGGATAAAGCCAGAAAGAGCCGTCGTATCTCCCTGGAATTCATGCTTGAGCAGGCGAACAAATTCGCTAACCACAAAGCCATCTGGTTTCCGTATAATATGGATTGGCGTGGTCGTGTTTACGCGGTATCGATGTTCAACCCGCAGGGTAATGACATGACCAAGGGTTTGTTGACCTTGGCCAAAGGTAAACCGATTGGCAAAGAAGGTTACTATTGGTTGAAGATCCACGGTGCGAACTGCGCAGGGGTGGATAAGGTACCGTTCCCGGAACGTATTAAATTCATCGAGGAAAACCACGAAAACATTATGGCGTGCGCAAAGAGCCCGCTGGAAAACACGTGGTGGGCAGAGCAGGACAGTCCATTCTGCTTCTTGGCGTTCTGTTTCGAATATGCAGGTGTCCAGCACCACGGCCTGAGCTATAACTGCAGCCTGCCGCTGGCCTTTGATGGTTCTTGTAGCGGTATTCAACACTTTAGCGCCATGCTGCGCGATGAGGTTGGTGGCAGAGCAGTGAATCTGCTGCCAAGCGAAACCGTGCAAGATATCTATGGCATTGTGGCTAAGAAGGTTAATGAAATCCTGCAAGCAGACGCGATTAACGGCACGGACAACGAGGTTGTCACCGTGACCGATGAAAACACCGGTGAGATCTCGGAGAAAGTGAAACTGGGCACCAAAGCCTTGGCTGGCCAATGGCTGGCATACGGCGTTACTCGCTCCGTGACCAAGCGTTCGGTGATGACCCTTGCTTACGGCAGCAAAGAGTTTGGTTTCCGCCAACAGGTTCTGGAGGATACTATCCAACCGGCCATCGACTCTGGTAAAGGCCTGATGTTCACGCAGCCGAATCAGGCAGCGGGCTATATGGCGAAGCTGATTTGGGAATCGGTTAGCGTTACCGTTGTTGCAGCGGTGGAGGCTATGAACTGGCTGAAATCTGCGGCGAAGCTGTTAGCTGCGGAGGTGAAGGACAAGAAGACGGGCGAAATCCTGCGTAAGCGCTGCGCAGTGCATTGGGTTACGCCGGATGGTTTTCCGGTATGGCAGGAGTACAAAAAGCCTATTCAGACCCGTCTGAACCTGATGTTTCTGGGTCAATTTCGTCTGCAGCCGACGATCAACACCAATAAGGACTCCGAGATCGACGCGCACAAACAGGAGTCTGGCATCGCACCCAATTTCGTTCACTCACAGGACGGTTCCCATCTGCGTAAGACCGTCGTGTGGGCACACGAGAAGTATGGCATTGAGTCGTTTGCATTGATCCACGATTCCTTTGGTACCATCCCGGCGGACGCTGCGAACCTGTTTAAAGCGGTTCGCGAAACCATGGTTGACACCTATGAAAGTTGCGATGTTCTGGCAGACTTTTATGATCAGTTCGCCGACCAACTGCATGAGTCCCAGCTGGATAAAATGCCGGCGTTACCGGCGAAGGGCAACTTAAATCTCCGCGACATCCTGGAATCTGATTTCGCCTTCGCGTAA

Amino acids sequence:

MNTINIAKNDFSDIELAAIPFNTLADHYGERLAREQLALEHESYEMGEARFRKMFERQLKAGEVADNAAAKPLITTLLPKMIARINDWFEEVKAKRGKRPTAFQFLQEIKPEAVAYITIKTTLACLTSADNTTVQAVASAIGRAIEDEARFGRIRDLEAKHFKKNVEEQLNKRVGHVYKKAFMQVVEADMLSKGLLGGEAWSSWHKEDSIHVGVRCIEMLIESTGMVSLHRQNAGVVGQDSETIELAPEYAEAIATRAGALAGISPMFQPCVVPPKPWTGITGGGYWANGRRPLALVRTHSKKALMRYEDVYMPEVYKAINIAQNTAWKINKKVLAVANVITKWKHCPVEDIPAIEREELPMKPEDIDMNPEALTAWKRAAAAVYRKDKARKSRRISLEFMLEQANKFANHKAIWFPYNMDWRGRVYAVSMFNPQGNDMTKGLLTLAKGKPIGKEGYYWLKIHGANCAGVDKVPFPERIKFIEENHENIMACAKSPLENTWWAEQDSPFCFLAFCFEYAGVQHHGLSYNCSLPLAFDGSCSGIQHFSAMLRDEVGGRAVNLLPSETVQDIYGIVAKKVNEILQADAINGTDNEVVTVTDENTGEISEKVKLGTKALAGQWLAYGVTRSVTKRSVMTLAYGSKEFGFRQQVLEDTIQPAIDSGKGLMFTQPNQAAGYMAKLIWESVSVTVVAAVEAMNWLKSAAKLLAAEVKDKKTGEILRKRCAVHWVTPDGFPVWQEYKKPIQTRLNLMFLGQFRLQPTINTNKDSEIDAHKQESGIAPNFVHSQDGSHLRKTVVWAHEKYGIESFALIHDSFGTIPADAANLFKAVRETMVDTYESCDVLADFYDQFADQLHESQLDKMPALPAKGNLNLRDILESDFAFA
